# A marine sponge associated strain of *Bacillus subtilis* and other marine bacteria can produce anticholinesterase compounds

**DOI:** 10.1186/1475-2859-13-24

**Published:** 2014-02-15

**Authors:** Sony Pandey, Ayinampudi Sree, Dipti Priya Sethi, Chityal Ganesh Kumar, Sudha Kakollu, Lipsa Chowdhury, Soumya Suchismita Dash

**Affiliations:** 1 Environment and Sustainability Department, CSIR - Institute of Minerals and Materials Technology, Bhubaneswar 751 013, India; 2 Medicinal Chemistry and Pharmacology Division, CSIR- Indian Institute of Chemical Technology, Uppal Road, Hyderabad 500 007, India

**Keywords:** Acetylcholinesterase inhibitors, Galanthamine, Alzheimer’s disease, *Fasciospongia cavernosa*, Marine bacteria, Screening, Soft coral, marine sediment

## Abstract

**Background:**

Acetylcholinesterase (AChE) inhibitors or anticholinesterases reduce the activity of enzyme acetylcholinesterase that degrades the neurotransmitter acetylcholine in the brain. The inhibitors have a significant pharmacological role in neurodegenerative diseases like Alzheimer’s and Parkinson’s etc. Although plants have been a significant source of these compounds, there are very few sporadic reports of microorganisms producing such inhibitors. Anticholinesterase activity in bacterial associates of marine soft corals and sponges were not previously reported.

**Results:**

We screened 887 marine bacteria for the presence of acetylcholinesterase inhibitors, in a microplate based assay, and found that 140 (15.8%) of them inhibit the electric eel enzyme, acetylcholinesterase. Majority of the active isolates were bacterial associates of soft corals followed by sediment isolates while most of the potent inhibitors belonged to the bacterial associates of marine sponges. Maximum inhibition (54%) was exhibited by a bacterial strain M18SP4P (ii), isolated from the marine sponge *Fasciospongia cavernosa*. Based on phenotypic characterization and 16S rDNA sequencing, the strain was identified as *Bacillus subtilis* - revealing yet another activity in a strain of the model organism that is considered to be a cell factory. TLC bioautography of the methanol extract of this culture, showed the presence of two major components having this activity, when compared to Galanthamine, the positive control.

**Conclusion:**

From the results of our study, we conclude that acetylcholinesterase inhibitors are quite prevalent in marine bacteria, particularly the bacterial associates of marine invertebrates. Several potential AChE inhibitors in marine bacteria are waiting to be discovered to provide easily manipulable natural sources for the mass production of these therapeutic compounds.

## Introduction

Acetylcholinesterase inhibitors (AChEIs) work by increasing the concentration of acetylcholine (ACh) in the brain, due to reduction in the rate of its breakdown by acetylcholinesterase (AChE). These inhibitors, naturally found in venoms, have a range of applications from pharmaceuticals to insecticides and weapons like nerve gas [1]. An important therapeutic use of AChE inhibitors is in the treatment of dementia associated with Alzheimer’s disease. There is no cure yet for this disease; four out of five FDA approved drugs are AChE inhibitors, which provide symptomatic relief, by improving signal transfer in the brain and slowing down the neurofibrillary degeneration [2]. However, the importance of acetylcholinesterase inhibitors cannot be underestimated because recent research shows that these inhibitors protect brain cells against free radical injury and β-amyloid toxicity in Alzheimer’s disease (AD) [3]. Despite the quest for new treatments to stop, slow or prevent AD, researchers around the world are looking for new sources of AChE inhibitors also, because of the concerns around the bioavailability and side-effects issues, associated with the existing, mostly synthetic, drugs for AD.

The natural sources are favoured for screening AChE inhibitors since they present enormous possibilities of discovering novel chemical structures with better properties. Majority of AChE inhibitors are derived from plants [1,4], but they were also isolated from extracts of some algae, fungi, cyanobacteria, marine phytoplankton and marine sessile organisms like sponges and soft corals. For example, anticholinesterase compounds like the arisugacin, sporothrin and curvularin have been isolated from fungi [5-7]; sargaquinoic acid was found in marine alga *Sargassum sagamianum*[8] and recently, biruloquinone isolated from a lichen forming fungi *Cladonia macilenta*, was identified as an inhibitor of acetylcholinesterase [9].

Very few AChE inhibitor compounds have been isolated from the bacterial sources. AChEI activity in bacteria was first reported in *Streptomyces antibioticus*, when two related furo-dioxa-phosphepin organophosphorus compounds, used in pesticides, were isolated from its cultures [10]. Subsequently, another AChE inhibitor, cyclophostin was found in *Strepomyces lavendulae*[11]. In the recent past, marinoquinoline A (a new alkaloid possessing pyrroloquinoline skeleton) was isolated from a marine gliding bacterium *Rapidithrix thailandica* (phylum Bacteroidetes). The AChE inhibitory activity of marinoquinoline A was discovered, after the compound was found to be structurally related to tacrine, which is an AChE inhibitor [12]. Marinoquinoline A was again isolated from another novel marine Bacteroidetes member *Ohtaekwangia kribbensis*[13]; hence this organism may, as well, inhibit AChE.

The latest discoveries about marinoquinoline A, coupled with, the reports claiming novel inhibitors from chemical investigations of marine sessile organisms [14-16], strongly indicate the potential of bacteria as a source of these important compounds from marine samples. Considering the enormous diversity of marine microorganisms, we set out to find the prevalence of acetylcholinesterase inhibitors in marine bacteria isolated from different samples of marine sponges, soft corals and sediments, using a high throughput microplate based assay. In this research report, we present an interesting finding that uncovers another activity - AChE inhibition - of a robust microorganism, amenable to manipulation and biotechnological scale-up for the production process.

## Results

### Screening assay

**Figure 1:**
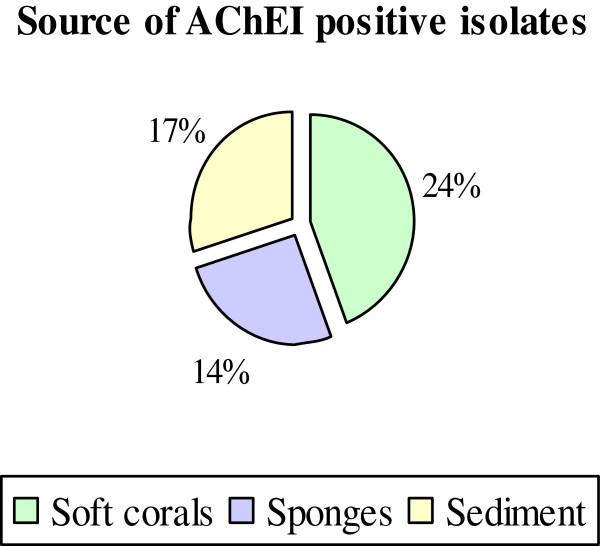
The distribution of AChEI positive isolates among soft corals, sponges and sediment.

**Figure 2:**
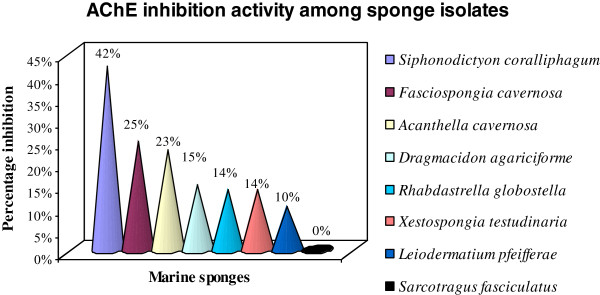
Comparison of AChE inhibition activity among sponge isolates.

Out of the 887 isolates tested for AChE inhibition activity, 140 isolates were found to be positive (Table 1; details in Additional file 1: Table S1). The proportion of positives hits was more in soft corals (n = 16 out of 66) followed by sediment (n = 53 out of 321) and sponges (n = 71 out of 500) as shown in Figure 1. The positive control, 0.1 μM galanthamine, inhibited 74% AChE while at higher concentrations, it showed 100% inhibition. Figure 2 shows a comparison of AChE inhibition activity of the eight sponge isolates taken in this study. Among the sponge samples *Siphonodictyon coralliphagum* isolates were the most active and potent inhibitors, followed by *Fasciospongia cavernosa, Acanthella cavernosa*, *Dragmacidon agariciforme, Rhabdastrella globostellata*, *Xestospongia testudinaria* and *Leiodermatium pfeifferae*; however, none of the 77 isolates of *Sarcotragus fasciculatus* were active. The sponge isolates have a comparatively large number of potent inhibitors, i.e, they showed higher percentages of inhibition (Additional file 1: Table S1). An isolate of the sponge *Fasciospongia cavernosa*, strain M18SP4Q (ii), showed the maximum percentage (54%) of AChE inhibition.

**Table 1:** AChE inhibition activity in marine isolates

**Sl. no.**	**Isolation source**	**No. of isolates screened**	**No. of active isolates**	**Percentage of active isolates (%)**	**No. of isolates showing >10% AChE inhibition activity**
1.	*Acanthella cavernosa*	39	9	23	0
2.	*Dragmacidon agariciforme*	110	17	15	4
3.	*Fasciospongia cavernosa*	8	2	25	1
4.	*Leiodermatium pfeifferae*	72	7	10	0
5.	*Rhabdastrella globostellata*	127	18	14	2
6.	*Siphonodictyon coralliphagum*	31	13	42	12
7.	*Xestospongia testudinaria*	36	5	14	4
8.	*Sarcotragus fasciculatus*	77	0	0	0
9.	Soft coral	66	16	24	2
10.	Sediment	321	53	17	26
	**Total**	**887**	**140**		**51**

### Identification of M18SP4Q (ii)

**Figure 3:**
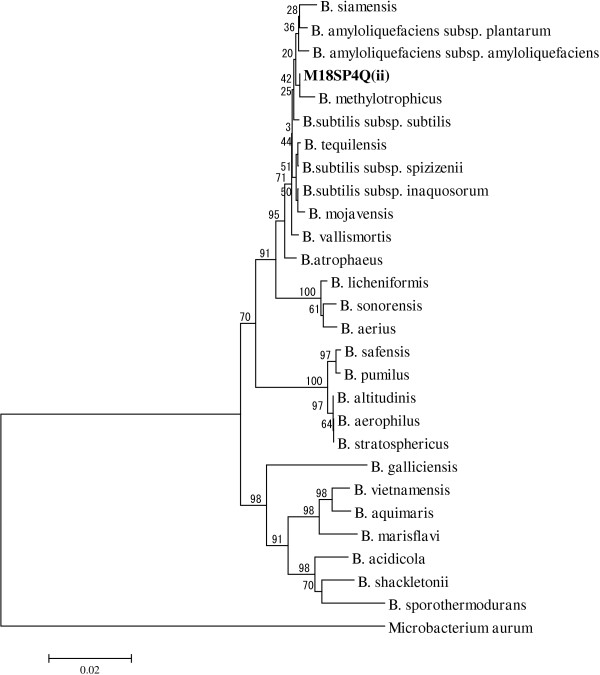
**The evolutionary relationship of the strain M18SP4Q (ii) with the 16S rRNA gene sequences of its closely related strains is shown in the neighbour joining phylogenetic tree.** The evolutionary history was inferred using the Neighbor-Joining method [17]. The optimal tree with the sum of branch length = 0.33445952 is shown. The percentage of replicate trees in which the associated taxa clustered together in the bootstrap test (1000 replicates) is shown above the branches [18]. The tree is drawn to scale, with branch lengths in the same units as those of the evolutionary distances used to infer the phylogenetic tree. The evolutionary distances were computed using the p-distance method [19] and are in the units of the number of base differences per site. The analysis involved 28 nucleotide sequences. All ambiguous positions were removed for each sequence pair. There were a total of 1539 positions in the final dataset.

The bacterial strain M18SP4Q (ii) was Gram positive slender rod with the spore in non-bulging sporangia. The strain was aerobic, grew in the presence of 6.5% NaCl and tested positive for starch, VP and Citrate; details are available in Table 2. The 16S rDNA sequence (GenBank accession number KC886741) of this strain showed 99.86% similarity with the type strain of *Bacillus subtilis* subsp. *subtilis*. The phenotypic characters combined with 16S rRNA gene sequence confirmed the identity of the strain as *Bacillus subtilis*. The evolutionary relationship of this strain M18SP4Q (ii), with the 16S rRNA gene sequences of its closely related strains is shown in the Neighbor-Joining phylogenetic tree (Figure 3).

**Table 2:** Biochemical characterization data of M18SP4Q (ii)

**Tests**	**M18SP4Q (ii)**
Colony morphology	
Configuration	Circular
Margin	Rhizoid
Elevation	Flat
Surface	Pale
Pigment	-
Opacity	Opaque
Gram’s reaction	Positive
Cell shape	Rods
Arrangement	Singles & pairs
Spore (s)	Present
Endospore	+
Position	Subterminal
Shape	Oval
Sporangia bulging	No
Motility	+
Temperature requirement	15 to 40°C
pH requirement	6-8
Growth at 6.5% NaCl	+
Growth under anaerobic condition	-
Voges Proskauer test	+
Citrate utilization	+
Starch hydrolysis	+
Nitrate reduction	+
Catalase test	+
Oxidase test	-

### TLC bioautography of M18SP4Q (ii)

**Figure 4:**
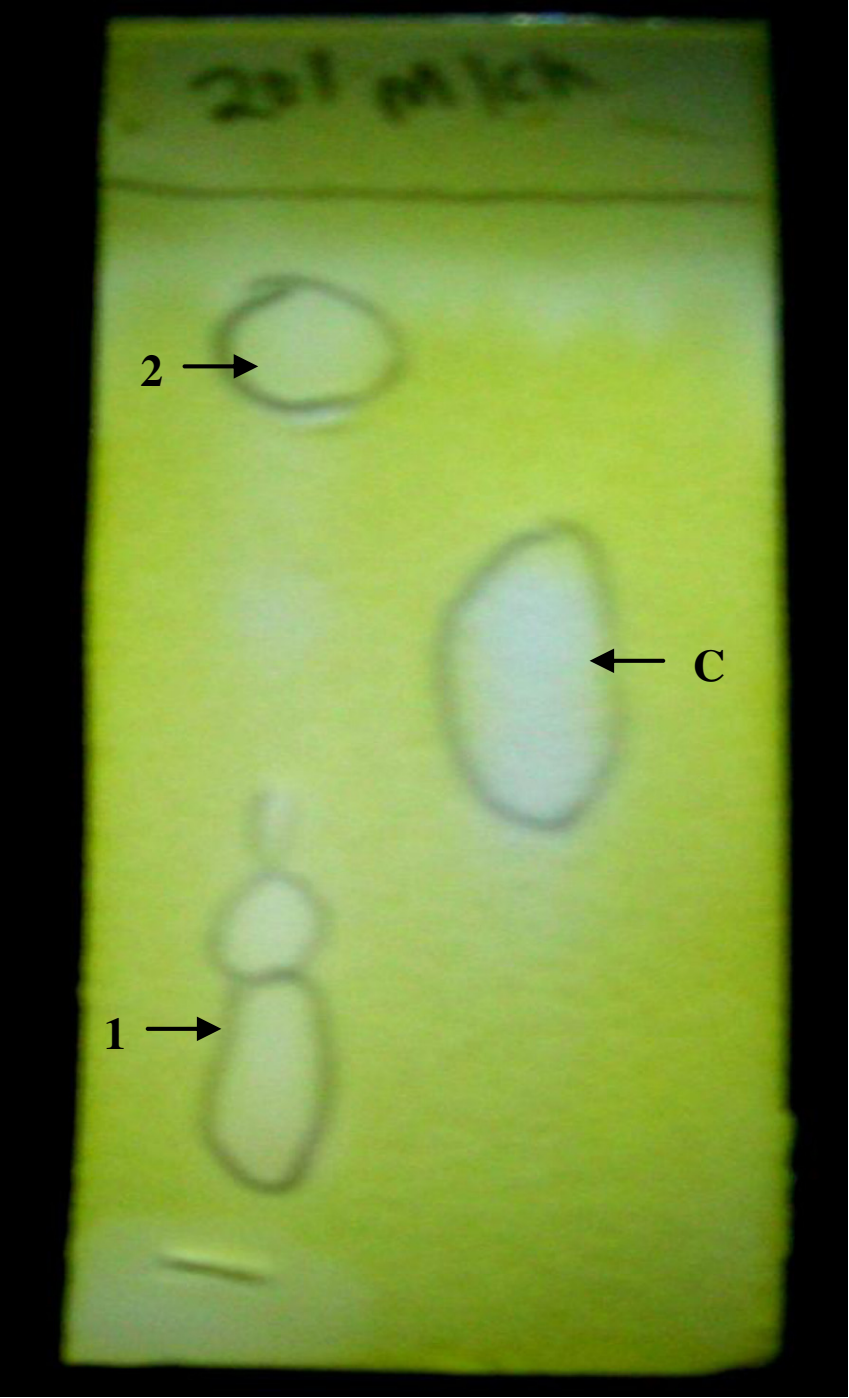
TLC bioautography of IMM46 extract showing two compounds 1 & 2 containing the inhibitor of acetylcholinesterase, and C - control (0.1 μM Galanthamine).

TLC bioautography, performed to analyze the inhibitor compound in the crude extract of M18SP4Q (ii) (extract code IMM 46), showed the presence of two major compounds at *Rf* – 0.45 and 0.85 in IMM46, while the galanthamine spot was observed at *Rf* – 0.58; indicating that the compounds are chemically different from galanthamine (Figure 4).

## Discussion

Many prominent researchers have reviewed the literature on marine natural products and unequivocally hailed the bioactive potential of marine microbes. Some of them proposed and demonstrated that marine invertebrate associated microbes are the primary producers of biologically active metabolites and play an important role in the host defense [20,21]. Nonetheless, very few studies have identified the microbial producers of sponge-associated compounds; on the other hand, few microorganisms of the diverse microbial community associated with the sponges have a known function. Although bridging this gap is not so easy for a single research group, we initiated a basic search for a function, i.e., acetylcholinesterase inhibition, in the diverse microbial associates of sponges, sediment and soft corals. This research report is the first account of AChE inhibition activity in the microbial associates of the marine invertebrates and sediment.

### Screening results analysis

Our findings showed a significant number, constituting 16%, of the isolates screened in this study with AChE inhibition activity. The major share of the positive cultures comes from soft corals followed by the sediment and sponge samples (Figure 1). Literature reviews of natural product AChE inhibitors shows that the majority of AChEIs are alkaloids followed by mono-, di-, tri- and sesqui-terpenes; while a few flavonoids, coumarins and other compounds are also on the list [22]. Soft corals are known to produce different types of terpenes; as a recent example, Bonnard et al. [14] discovered novel bioactive natural terpenes upon chemical investigations of comorian soft corals. There are some studies showing coral-associated microorganisms as the biosynthetic source of terpene derivatives; like, the dinoflagellate *Symbiodinium sp.* was identified as a producer of pseudopterosins, the pharmacologically important diterpene glycosides, in a gorgonian soft coral [23]. Although these findings are not connected, it is apparent that the AChE inhibiting terpenes have been found in different soft coral extracts, and coral microbial symbionts too have been shown to produce a class of terpenes. A very recent study, using the new technique of “genome mining” [24], also reveals tremendous biosynthetic potential of several bacterial species for terpene synthesis. Hence, it may be assumed that the terpenes isolated from different soft coral may have originated in the associated microbes.

Other chemical compounds from invertebrate extracts may show similar links if studied in depth because literature is replete with reports which show the involvement of different chemical classes of compounds in many of the biological activities. If such different classes of compounds can show the same activity, the chances of these compounds being found in the microbial world are not surprising. It has been observed in chemical investigations of natural extracts that the novel compounds are mostly variants or derivatives of existing major chemical classes of compounds. So, the general opinion of microbes being the primary producers of sponge or coral metabolites may gather more proofs with the concerted efforts of marine microbiologists and natural product chemists. This also explains why such large numbers of marine isolates exhibited AChEI activity.

### AChEIs in sponge microbes

An examination of Table 1 shows that the maximum number of active isolates belong to the sponge *Siphonodictyon coralliphagum*, exhibiting consistently higher percentages of inhibition as compared to all other isolates. Earlier, a number of new meroterpenoid and spirosesquiterpene aldehyde compounds, like corallidictyals and liphagal; siphonodictyal sulfate and akadisulfates, have been isolated from the extracts of the sponge *Siphonodictyon coralliphagum* (= *Aka coralliphaga*); which showed protein kinase C inhibition, PI3K inhibitory activity and radical-scavenging activity respectively [25-27]. Some siphonodictyals and corallidictyals have also been reported to have antimicrobial activity [28,29]. The other sponges from which compounds with antimicrobial activity have been isolated are *Fasciospongia cavernosa*[30]*and Acanthella cavernosa*[31-33]. A careful study of the previous literature showed that several compounds with AChEI activity, isolated from the sponges, mostly derivatives of alkaloids and terpenes [22]. However, anticholinesterase activity of the compounds of the above mentioned sponges or their microbial associates was not known so far, which may be due to limited research in this area. Recently, a web resource - DESMSCI – was developed for knowledge generation on marine sponge compounds interactions including a useful “hypothesis generation” feature [34]. Using this web resource, the authors could generate and theoretically validate a hypothesis, by linking terms from different dictionaries. According to this hypothesis there is a possibility of finding a novel mode of action for a sponge compound variolin (an alkaloid) in AD. Thus, it is apparent that sponge compounds, like alkaloids, may not just inhibit AChE to provide symptomatic relief in AD, but may even have a disease modifying effect which needs to be studied in depth.

### AChEI in *Bacillus subtilis*

The isolate M18SP4Q (ii), which showed the maximum percentage of AChE inhibition in the present screening, was taxonomically identified as *Bacillus subtilis*. This omnipresent saprophytic bacterium can survive in extreme conditions and is ecologically important due to its ability to produce a wide variety of enzymes; as a result, they play varied role from nutrient recycling to heavy metal accumulation or detoxification. Besides enzymes, they also produce antibiotics and their biosurfactant lipopeptides, called surfactins, have been reported to have mosquito larvicidal and pupicidal activity [35-37]. It is also commercially used in the production of amylase, protease, lipase, amino acids, inosine, ribosides, hyaluronic acid and polyhydroxybutarate. *Bacillus subtilis* is a favorable workhorse for the biotechnology industry because it is highly responsive to genetic manipulation. Recently, van Dijl and Hecker [38] suggested that *Bacillus subtilis* can be engineered into next-generation super-secreting cell factory using combined systems and synthetic biology approach. Thus, the detection of acetylcholinesterase inhibition in a *Bacillus subtilis* strain is certainly a significant finding.

### Diversity of bacteria with AChEI activity

Microbes producing AChE inhibitors are few and far between in the past literature. In our taxonomic investigation of some marine bacteria, we found several different genera of bacteria produce AChE inhibitors, like*, Psychrobacter, Microbacterium, Stenotrophomonas, Planococcus, Nocardia, Streptomyces sp., Leucobacter, Bacillus sp., Virgibacillus and Brevibacterium sp*. (Additional file 2: Table S2), apart from the *Bacillus subtilis* strain. Involvement of diverse groups of microorganisms in AChE inhibitor production increases the chances of finding structurally unique metabolites. Recently, phylogenetically diverse urea utilizing microbes associated with the sponge *Xestospongia testudinaria* was reported, and our group has also reported a diverse marine microbial community producing beta-glucosidase inhibitors [39,40]. The research on marine bacteria has not been extensive; therefore, the bioactive strains isolated from marine samples may present themselves as new to natural products research. Thus, the efforts to screen natural products from marine bacteria for industrially important activities are desirable conjointly with the molecular techniques in the metagenomic era.

## Conclusion

Acetylcholinesterase inhibitors in bacteria have not received adequate attention of the researchers. Our assumption on the preponderance of AChEIs in marine bacteria proved to be correct. The AChEI activity has been detected in good numbers, in the microbial associates of soft corals, sponges and sediments. *Bacillus subtilis* - a well studied and exploited species - presented yet another activity, though it remains to be seen whether the compound responsible for this activity is new or known. These results imply that many anticholinesterase compounds are waiting to be discovered in marine bacteria of different lineages. Chemical investigation of hits from this study may be pursued in order to use these compounds. In future, the marine microbial secondary metabolites may be considered for further studies in the development of drugs for the treatment of AD because the present work shows a significant number of marine bacteria possess anticholinesterase activity.

## Materials and methods

### Chemicals used

AChE Type VI-S, EC 3.1.1.7, from electric eel (*Electrophorus electricus*) and galanthamine hydrobromide from *Lycoris* sp. were purchased from Sigma (St. Louis, MO, USA). Acetylthiocholine iodide (ATCI), 5,5’-dithiobis-(2-nitrobenzoic acid) (DTNB), bovine serum albumin (BSA), dimethylsulfoxide (DMSO), Tris–HCl and microbiological media were procured from HiMedia Laboratories Pvt. Ltd., Mumbai, India. The chemicals used in the study were of the highest purity and used without further purification.

### Buffers and reagents

A stock solution of 1.17 mg/ml acetylcholinesterase (425.94 U/mg) was prepared with 0.1% BSA in buffer A (50 mM Tris–HCl, pH-8) and stored in −20°C, and working solutions of 0.01 U/ml and 3 U/ml were prepared by dilution. Phosphate buffer (pH 7.5), 1.5 mM ATCI reconstituted in phosphate buffer and 3 mM DTNB in phosphate buffer. Galanthamine 5 mg/ml was dissolved in methanol and further diluted in phosphate buffer to make a working stock of 0.1 μM ml^-1^.

### Bacterial strains and culture conditions

A large number of marine microorganisms were isolated from the samples of marine sponges, soft corals and sediment, collected from the Bay of Bengal on the east coast of India. The initial isolations were carried out on a variety of media prepared in seawater at temperatures ranging from 20-30°C. Many of these isolates were screened for the investigation of the presence of acetylcholinesterase inhibitors in the associates of marine sponges, soft corals and sediment samples. In this study, a total of 887 marine microorganisms isolated from samples of 8 sponges [total n = 500; *Rhabdastrella globostella* (n = 127), *Acanthella cavernosa* (n = 39), *Dragmacidon agariciforme* (n = 110), *Leiodermatium pfeifferae* (n = 72), *Fasciospongia cavernosa* (n = 08), *Xestospongia testudinaria* (n = 36), *Siphonodictyon coralliphagum* (n = 31) and *Sarcotragus fasciculatus* (n = 77)], 2 unidentified soft corals (n = 66) and 5 sediments (n = 321) were investigated. All these organisms grew on Nutrient Agar media (HiMedia, Mumbai) prepared in 50% aged natural seawater at 30°C within 48–72 hrs.

### Extraction of metabolites

The microbial metabolites were extracted as described in Pandey et al. [40]. Briefly, the cultures were grown in 50 ml of Nutrient Broth with 50% seawater, at 30°C/48 hrs and 200 rpm in a rotary shaker, followed by centrifugation at 9000 rpm/20 min to remove the cell mass. The supernatant was mixed with 10% Diaion HP-20 (Sigma) resin for 30 min on a magnetic stirrer. Then the Diaion resin along with supernatant was packed in a glass column, washed with 15 ml distilled water, and eluted with 20 ml methanol. The collected methanol was evaporated, and the extract was dissolved in DMSO.

### AChE inhibition assay

AChE inhibitory activity was tested using a microplate assay based on the modified method of Ellman et al. [41]. The enzyme hydrolyzed the substrate acetylthiocholine resulting in the product thiocholine which reacts with Ellman’s reagent (DTNB) *in situ* and gives the yellow coloured chromophore of 5-thio-2-nitrobenzoic acid (TNB), which can be detected at 405 nm. The absorption intensity of TNB adduct (405 nm) is proportional to the formation of thiocholine, therefore, the AChE activity. In each well of the 96-well microtiter plate, 5 μl of microbial extract and 10 μl of 0.01 U ml^-1^ of acetylcholine esterase were incubated at 4°C for 20 min before the addition of 10 μl of 1.5 mM ATCI in phosphate buffer (pH 7.5), 60 μl of 3 mM DTNB in phosphate buffer (pH 7.5), 60 μl of 0.1 M phosphate buffer (pH 7.5) and absorbance was measured at 405 nm every 30 s for 4 min × 8 times on a TRIAD multimode reader (Dynex Technologies, Inc., Chantilly, VA). The rate of reactions was calculated using the Manager software of the multimode reader. Percentage of inhibition was calculated by comparing the rates for each sample with respect to the blank (10% DMSO in buffer). Galanthamine hydrobromide (0.1 μM ml^-1^) in DMSO solvent was used as a positive control. Each assay was performed in triplicate, and the AChE inhibitory values are the average of three independent experiments. The percent inhibition was calculated using the following equation:

$$\%\;\mathrm{Inhibition}=\frac{A_{\mathrm{control}}–A_{\mathrm{sample}}}{A_{\mathrm{control}}}\times 100$$

where A_sample_ was the absorbance of the sample microbial extract, and A_control_ was the absorbance of the blank reaction mixture without the inhibitor - negative control.

### TLC bioautography

TLC bioautographic assay was carried out for the crude extract (IMM46), from the isolate M18SP4Q (ii) that showed the highest inhibition of AChE, as described by Rhee et al. [42], but with some modifications in the reagent concentrations. The substrate ATCI and chromogen DTNB was prepared as 2 mM solutions in 10 ml of buffer A. TLC assay was done on Silicagel 60 F 254 nm plates of 0.2 mm thickness with 1 μL each of M18SP4Q (ii) extract (100 mg/ml) and 1 μL of 0.1 μM ml^-1^ galanthamine spotted on the plate, developed in chloroform:methanol (8:2) in the mobile phase. After the TLC plate was developed, equal volume of 2 mM solutions of ATCI and DTNB was sprayed on the TLC plate until the plate was saturated. The plate was air dried for 5 min and then the enzyme - 10 ml of 3 U/ml AChE enzyme solution - was sprayed on the TLC plate. Yellow colour appeared within 5–10 min on the TLC plate except in places where the inhibitor was present.

### Microbial identification

The isolate, M18SP4Q (ii), was identified by partial 16S rRNA gene sequencing and phenotypic characterization [43]. The colony and cell morphology, including spores, were characterized, and starch, nitrate, VP and NaCl tolerance tests were performed as described by Claus and Berkeley [44]. The 16S rRNA gene based identification was done as described in Pandey et al. (36). Briefly, a 25 μl PCR reaction was set up with 10 pmol primers – 27 F and 1492R, 150 ng DNA, 5 mM dNTPs, 1× Taq polymerase buffer containing 15 mM MgCl_2_ and 0.5 μl of Taq DNA polymerase (5 U/μl, Fermentas) as per the programme: 94°C for 5 min, 30 cycles of 94°C for 1 min, 50°C for 1 min, 72°C for 2 min and 72°C for 10 min. The PCR product was separated on 1% agarose gel, and the DNA fragments were extracted. The purified 16S rDNA was sequenced in Beckman Coulter Ceq 8000 genetic analysis system. The sequence thus obtained was deposited in GenBank and aligned with the other sequences using BLAST program in NCBI (http://blast.ncbi.nlm.nih.gov). The EzTaxon server 2.1 (http://eztaxon-e.ezbiocloud.net/) was also used to obtain the closest matching type strain sequences from the database. The identity of the strain was established based on the phenotypic characters and sequence identity.

### Phylogenetic analysis

The 16S rDNA sequences of the closest relatives (type strains) of the strain M18SP4Q (ii) were retrieved from EzTaxon, and multiple sequence alignment was performed using Clustal_X program [45]. The evolutionary relatedness of the strain was inferred using the Neighbor-Joining method, distance calculated using the p-distance method, followed by bootstrap test in 1000 replicates for each cluster and the phylogenetic tree was drawn in *MEGA5.0* program available at http://www.megasoftware.net/[46].

## Competing interests

The authors declare no competing interests.

## Authors’ contributions

SP contributed to the design of experiments, acquisition, analysis and interpretation of data, and drafted the manuscript. AS conceived and designed the work on marine microbes and anticholinesterase inhibitors, collected marine sponge, soft coral and sediment samples and helped in editing of the manuscript. CGK contributed to the editing of the manuscript, design of experiments related to anticholinesterase screening assays, acquisition and interpretation of data. DPS, SK, LC and SSD helped in execution of experimental work and acquisition of data. All authors have read and approved the final manuscript.

## Supplementary Material

Additional file 1: Table S1 Percentage of AChE inhibition in extracts of marine isolates. Click here for file

Additional file 2: Table S2 Identity of some AChEI positive strains based on morphological characters and 16S rRNA gene sequence match. Click here for file
