# Supplementary material for: A marine sponge associated strain of Bacillus subtilis and other marine bacteria can produce anticholinesterase compounds
Source: Microb Cell Fact. 2014 Feb 15;13:24. doi: 10.1186/1475-2859-13-24 (PMC3932841; doi:10.1186/1475-2859-13-24)
Supplement: Additional file 1: Table S1 — Percentage of AChE inhibition in extracts of marine isolates. [file 1475-2859-13-24-S1.doc]

**Additional file 1: Table S1. Percentage of AChE inhibition in extracts of** marine isolates

| Sl no. | Isolation source & year of collection | Extract code | AChE activity |
| --- | --- | --- | --- |
| 1 | *Acanthella cavernosa* | IMM 214 | 3% |
| 2 | *Acanthella cavernosa* | IMM 215 | 7% |
| 3 | *Acanthella cavernosa* | IMM 217 | 3% |
| 4 | *Acanthella cavernosa* | IMM 235 | 7% |
| 5 | *Acanthella cavernosa* | IMM 239 | 4% |
| 6 | *Acanthella cavernosa* | IMM 240 | 3% |
| 7 | *Acanthella cavernosa* | IMM 251 | 9% |
| 8 | *Acanthella cavernosa* | IMM 252 | 3% |
| 9 | *Acanthella cavernosa* | IMM 255 | 4% |
| 10 | *Dragmacidon agariciforme* | IMM 139 | 3% |
| 11 | *Dragmacidon agariciforme* | IMM 150 | 7% |
| 12 | *Dragmacidon agariciforme* | IMM 405 | 5% |
| 13 | *Dragmacidon agariciforme* | IMM 406 | 22% |
| 14 | *Dragmacidon agariciforme* | IMM 409 | 2% |
| 15 | *Dragmacidon agariciforme* | IMM 410 | 8% |
| 16 | *Dragmacidon agariciforme* | IMM 420 | 22% |
| 17 | *Dragmacidon agariciforme* | IMM 437 | 14% |
| 18 | *Dragmacidon agariciforme* | IMM 460 | 6% |
| 19 | *Dragmacidon agariciforme* | IMM 472 | 3% |
| 20 | *Dragmacidon agariciforme* | IMM 473 | 8% |
| 21 | *Dragmacidon agariciforme* | IMM 478 | 3% |
| 22 | *Dragmacidon agariciforme* | IMM 58 | 8% |
| 23 | *Dragmacidon agariciforme* | IMM 69 | 6% |
| 24 | *Dragmacidon agariciforme* | IMM 82 | 10% |
| 25 | *Dragmacidon agariciforme* | IMM 85 | 8% |
| 26 | *Dragmacidon agariciforme* | IMM 92 | 9% |
| 27 | *Fasciospongia cavernosa* | IMM 43 | 8% |
| 28 | *Fasciospongia cavernosa* | IMM 46 | 54% |
| 29 | *Leiodermatium pfeifferae* | IMM 216 | 7% |
| 30 | *Leiodermatium pfeifferae* | IMM 238 | 9% |
| 31 | *Leiodermatium pfeifferae* | IMM 285 | 9% |
| 32 | *Leiodermatium pfeifferae* | IMM 303 | 9% |
| 33 | *Leiodermatium pfeifferae* | IMM 315 | 4% |
| 34 | *Leiodermatium pfeifferae* | IMM 316 | 6% |
| 35 | *Leiodermatium pfeifferae* | IMM 321 | 5% |
| 36 | Mangrove sediment | IMM 569 | 19% |
| 37 | Mangrove sediment | IMM 575 | 28% |
| 38 | Mangrove sediment | IMM 583 | 2% |
| 39 | Mangrove sediment | IMM 585 | 14% |
| 40 | Mangrove sediment | IMM 589 | 20% |
| 41 | Mangrove sediment | IMM 591 | 14% |
| 42 | Mangrove sediment | IMM 593 | 3% |
| 43 | Mangrove sediment | IMM 596 | 20% |
| 44 | Mangrove sediment | IMM 597 | 29% |
| 45 | Mangrove sediment | IMM 606 | 2% |
| 46 | Mangrove sediment | IMM 610 | 3% |
| 47 | Mangrove sediment | IMM 615 | 4% |
| 48 | Mangrove sediment | IMM 618 | 2% |
| 49 | Mangrove sediment | IMM 619 | 13% |
| 50 | Mangrove sediment | IMM 622 | 11% |
| 51 | Mangrove sediment | IMM 626 | 2% |
| 52 | Mangrove sediment | IMM 636 | 16% |
| 53 | Mangrove sediment | IMM 641 | 5% |
| 54 | Mangrove sediment | IMM 642 | 10% |
| 55 | Mangrove sediment | IMM 644 | 4% |
| 56 | Mangrove sediment | IMM 645 | 5% |
| 57 | Mangrove sediment | IMM 652 | 2% |
| 58 | Mangrove sediment | IMM 661 | 4% |
| 59 | Mangrove sediment | IMM 665 | 1% |
| 60 | Mangrove sediment | IMM 690 | 11% |
| 61 | Mangrove sediment | IMM 691 | 24% |
| 62 | *Rhabdastrella globostellata* | IMM 12 | 4% |
| 63 | *Rhabdastrella globostellata* | IMM 14 | 7% |
| 64 | *Rhabdastrella globostellata* | IMM 156 | 8% |
| 65 | *Rhabdastrella globostellata* | IMM 158 | 3% |
| 66 | *Rhabdastrella globostellata* | IMM 160 | 7% |
| 67 | *Rhabdastrella globostellata* | IMM 165 | 8% |
| 68 | *Rhabdastrella globostellata* | IMM 172 | 8% |
| 69 | *Rhabdastrella globostellata* | IMM 178 | 10% |
| 70 | *Rhabdastrella globostellata* | IMM 179 | 7% |
| 71 | *Rhabdastrella globostellata* | IMM 222 | 3% |
| 72 | *Rhabdastrella globostellata* | IMM 223 | 5% |
| 73 | *Rhabdastrella globostellata* | IMM 232 | 3% |
| 74 | *Rhabdastrella globostellata* | IMM 233 | 4% |
| 75 | *Rhabdastrella globostellata* | IMM 242 | 4% |
| 76 | *Rhabdastrella globostellata* | IMM 34 | 9% |
| 77 | *Rhabdastrella globostellata* | IMM 50 | 8% |
| 78 | *Rhabdastrella globostellata* | IMM 53 | 4% |
| 79 | *Rhabdastrella globostellata* | IMM 70 | 10% |
| 80 | Sediment | IMM 236 | 5% |
| 81 | Sediment | IMM 383 | 6% |
| 82 | Sediment | IMM 419 | 28% |
| 83 | Sediment | IMM 422 | 25% |
| 84 | Sediment | IMM 44 | 6% |
| 85 | Sediment | IMM 448 | 15% |
| 86 | Sediment | IMM 461 | 5% |
| 87 | Sediment | IMM 479 | 10% |
| 88 | Sediment | IMM 512 | 6% |
| 89 | Sediment | IMM 548 | 2% |
| 90 | Sediment | IMM 55 | 10% |
| 91 | Sediment | IMM 560 | 22% |
| 92 | Sediment | IMM 598 | 14% |
| 93 | Sediment | IMM 599 | 20% |
| 94 | Sediment | IMM 604 | 2% |
| 95 | Sediment | IMM 605 | 6% |
| 96 | Sediment | IMM 623 | 6% |
| 97 | Sediment | IMM 659 | 4% |
| 98 | Sediment | IMM 689 | 24% |
| 99 | Sediment | IMM 696 | 2% |
| 100 | Sediment | IMM 882 | 20% |
| 101 | Sediment | IMM 884 | 28% |
| 102 | Sediment | IMM 888 | 22% |
| 103 | Sediment | IMM 890 | 1% |
| 104 | Sediment | IMM 891 | 3% |
| 105 | Sediment | IMM 892 | 19% |
| 106 | Sediment | IMM 895 | 24% |
| 107 | *Siphonodictyon coralliphagum* | IMM 861 | 22% |
| 108 | *Siphonodictyon coralliphagum* | IMM 863 | 11% |
| 109 | *Siphonodictyon coralliphagum* | IMM 867 | 27% |
| 110 | *Siphonodictyon coralliphagum* | IMM 868 | 16% |
| 111 | *Siphonodictyon coralliphagum* | IMM 870 | 3% |
| 112 | *Siphonodictyon coralliphagum* | IMM 871 | 30% |
| 113 | *Siphonodictyon coralliphagum* | IMM 872 | 35% |
| 114 | *Siphonodictyon coralliphagum* | IMM 874 | 18% |
| 115 | *Siphonodictyon coralliphagum* | IMM 878 | 25% |
| 116 | *Siphonodictyon coralliphagum* | IMM 879 | 36% |
| 117 | *Siphonodictyon coralliphagum* | IMM 880 | 10% |
| 118 | *Siphonodictyon coralliphagum* | IMM 905 | 18% |
| 119 | *Siphonodictyon coralliphagum* | IMM 906 | 40% |
| 120 | Soft coral | IMM 250 | 6% |
| 121 | Soft coral | IMM 311 | 9% |
| 122 | Soft coral | IMM 313 | 6% |
| 123 | Soft coral | IMM 314 | 7% |
| 124 | Soft coral | IMM 318 | 5% |
| 125 | Soft coral | IMM 319 | 3% |
| 126 | Soft coral | IMM 320 | 6% |
| 127 | Soft coral | IMM 323 | 6% |
| 128 | Soft coral | IMM 324 | 8% |
| 129 | Soft coral | IMM 333 | 8% |
| 130 | Soft coral | IMM 376 | 39% |
| 131 | Soft coral | IMM 380 | 6% |
| 132 | Soft coral | IMM 381 | 3% |
| 133 | Soft coral | IMM 412 | 28% |
| 134 | Soft coral | IMM 436 | 4% |
| 135 | Soft coral | IMM 475 | 4% |
| 136 | *Xestospongia testudinaria* | IMM 774 | 30% |
| 137 | *Xestospongia testudinaria* | IMM 782 | 52% |
| 138 | *Xestospongia testudinaria* | IMM 799 | 7% |
| 139 | *Xestospongia testudinaria* | IMM 803 | 27% |
| 140 | *Xestospongia testudinaria* | IMM 804 | 23% |
