# Supplementary material for: A marine sponge associated strain of Bacillus subtilis and other marine bacteria can produce anticholinesterase compounds
Source: Microb Cell Fact. 2014 Feb 15;13:24. doi: 10.1186/1475-2859-13-24 (PMC3932841; doi:10.1186/1475-2859-13-24)
Supplement: Additional file 2: Table S2 — Identity of some AChEI positive strains based on morphological characters and 16S rRNA gene sequence match. [file 1475-2859-13-24-S2.doc]

**Additional file 2: Table S2. Identity of some AChEI positive strains based on morphological characters and 16S rRNA gene sequence match**

| **Sl no.** | **Strain designation** | **Extract code** | **AChE activity** | **Morphological characters** | **16S rRNA gene sequence percentage identity**  **as *Genus sp.* (Phylum)** |
| --- | --- | --- | --- | --- | --- |
| 1 | M21SP_3_PA_2_10^-5^(5) | IMM 53 | 4% | Cells - Gram positive, irregular slender rods, non-motile, aerobic, salt tolerant (15%), Colonies - light yellow, medium, circular, opaque and convex. | 97.06% *Brevibacterium sp*. (Actinobacteria) |
| 2 | M21SP3qD10-3(3) | IMM 160 | 7% | Cells – Gram positive filamentous rods, motile, facultative anaerobe, spores in swollen sporangia, slightly salt tolerant (5%); Colonies – Creamish light grey, circular, regular, low convex and opaque | 99.65% *Virgibacillus pantothenticus* (Firmicutes) |
| 3 | M21SD2A210-3(2) | IMM 236 | 5% | Cells - Gram negative, ovoid, non-motile, aerobic, grows at 4^o^C, Colonies - white, circular, smooth and convex. | 99.70% *Psychrobacter maritimus* (Gammaproteobacteria) |
| 4 | M21SD1C10-3(3b) | IMM 419 | 28% | Cells - Gram positive, irregular slender rods and cocci, without spores, non-motile, aerobic, salt tolerant (4%), Colonies - light yellow, medium, circular, smooth, translucent and convex. | 99.72% *Microbacterium oleivorans* (Actinobacteria) |
| 5 | M21SD1H10-5(2) | IMM 479 | 10% | Cells - Gram negative, slightly curved rods, non-sporing, non-motile, facultative anaerobic, slightly salt tolerant (4%); Colonies - Light yellow, medium, circular, entire, transleucent, slightly raised | 98.53% *Stenotrophomonas rhizophila* (Gammaproteobacteria) |
| 6 | M21SD1C10-5(5)(2) | IMM 548 | 2% | Cells - Gram positive, coccoid rods without spores, non-motile, aerobic, salt tolerant (2%), Colonies - light orange, smooth, circular, translucent and convex. | 98.67% *Microbacterium sp* (Actinobacteria)*.* |
| 7 | C1/2 S3 | IMM 569 | 20% | Cells - Gram positive, branching filaments fragmented to rods in substrate mycelium, aerial mycelium visible, non-motile, aerobic, slightly salt tolerant (4%); Colonies – Ochre yellow substrate mycelium, pinkish aerial mycelium, slightly raised | 99. 00% *Nocardia sienata* (Actinobacteria) |
| 8 | C3/1 S9 | IMM 593 | 4% | Cells - Gram positive, filamentous, spores in spirals; Colonies – White aerial mycelium; Reverse - White. | 99.78% *Streptomyces rochei* (Actinobacteria) |
| 9 | GDSMM1(3-i) | IMM 689 | 24% | Mycelia – Gram positive, filamentous, spores in spirals; Colonies – Large, circular, grey aerial mycelium in concentric rings around the depressed centre, some white spores in the colony. Reverse-Orange with dark centre. | 98% *Streptomyces coelicoflavus*  (Actinobacteria) |
| 10 | SP2Z/3(2) | IMM 861 | 22% | Cell – Gram positive rods, spores in non-swollen sporangia, motile, aerobic, salt tolerant (5%); Colonies - Pale milky white, large, opaque and rhizoid | 100% *Bacillus amyloliquefaciens subsp. amyloliquefaciens*  (Firmicutes) |
| 11 | SP2M1/2(1) | IMM 868 | 17% | Cells - Gram positive, central spores in non-bulging sporangia, aerobic; Colonies - Pale brownish white colour, regular, raised circular and smooth. | 99.41% *Bacillus tequilensis* (Firmicutes) |
| 12 | SP2Zd/5(6) | IMM 872 | 35% | Cells – Gram positive rods, motile, facultative anaerobe, spores in non-swollen sporangia, moderately salt tolerant (7%); Colonies - Brownish cream, finely rhizoid, slightly raised and opaque. | 98.05% *Bacillus firmus*(Firmicutes) |
| 13 | SP2Z/2(1) | IMM 878 | 25% | Cells - Gram positive cocci, aerobic, motile; Colonies - Orange, circular, small, regular margin, low convex, transparent, smooth surface. | 98.83% *Planococcus maitriensis* (Firmicutes) |
| 14 | SP2Zd/3(10) | IMM 880 | 10% | Cell – Gram positive rod, non-sporulating, aerobic, non-motile, salt tolerant (8%); Colonies - yellow, medium, entire, smooth, transleucent. | 99.67% *Leucobacter chromiiresistens* (Actinobacteria) |
| 15 | SP2M1/3(1) | IMM906 | 41% | Cell – Gram positive rods, non-bulging spores, motile, aerobic; Colonies - Brownish white, medium, irregular margin, shiny, raised and opaque. | 100% *Bacillus subtilis subsp. Spizizenii* (Firmicutes) |
| 16 | SP2Z/1(5) | IMM905 | 19% | Cell – Gram positive sporulating rods, motile, aerobic, salt tolerant (2%); Colonies - Brownish grey-white, medium, irregular margin, shiny and opaque. | 98.20% *Bacillus stratosphaericus*(Firmicutes) |
